# Evidence of synergy coordination patterns of upper-limb motor control in stroke patients with mild and moderate impairment

**DOI:** 10.3389/fphys.2023.1214995

**Published:** 2023-09-11

**Authors:** Kunkun Zhao, Chuan He, Wentao Xiang, Yuxuan Zhou, Zhisheng Zhang, Jianqing Li, Alessandro Scano

**Affiliations:** ^1^ School of Biomedical Engineering and Informatics, Nanjing Medical University, Nanjing, Jiangsu, China; ^2^ Department of Rehabilitation Medicine, The Affiliated Jiangsu Shengze Hospital of Nanjing Medical University, Suzhou, Jiangsu, China; ^3^ School of Mechanical Engineering, Southeast University, Nanjing, Jiangsu, China; ^4^ Institute of Systems and Technologies for Industrial Intelligent Technologies and Advanced Manufacturing, Italian Council of National Research, Milan, Italy

**Keywords:** electromyography (EMG), synergic patterns, muscle synergies, rehabilitation, stroke, upper-limb

## Abstract

**Objectives:** Previous studies showed that the central nervous system (CNS) controls movements by recruiting a low-dimensional set of modules, usually referred to as muscle synergies. Stroke alters the structure and recruitment patterns of muscle synergies, leading to abnormal motor performances. Some studies have shown that muscle synergies can be used as biomarkers for assessing motor function. However, coordination patterns of muscle synergies in post-stroke patients need more investigation to characterize how they are modified in functional movements.

**Methods:** Thirteen mild-to-moderate stroke patients and twenty age-matched healthy subjects were recruited to perform two upper-limb movements, hand-to-mouth movement and reaching movement. Muscle synergies were extracted with nonnegative matrix factorization. We identified a set of reference synergies (i.e., averaged across healthy subjects) and typical synergies (i.e., averaged across stroke subjects) from the healthy group and stroke group respectively, and extracted affected synergies from each patient. Synergy similarity between groups was computed and analyzed. Synergy reconstruction analysis was performed to verify synergy coordination patterns in post-stroke patients.

**Results:** On average, three synergies were extracted from both the healthy and stroke groups, while the mild impairment group had a significantly higher number of synergies than the healthy group. The similarity analysis showed that synergy structure was more consistent in the healthy group, and stroke instead altered synergy structure and induced more variability. Synergy reconstruction analysis at group and individual levels showed that muscle synergies of patients often showed a combination of healthy reference synergies in the analyzed movements. Finally, this study associated four synergy coordination patterns with patients: merging (equilibrium and disequilibrium), sharing (equilibrium and disequilibrium), losing, and preservation. The preservation was mainly represented in the mild impairment group, and the moderate impairment group showed more merging and sharing.

**Conclusion:** This study concludes that stroke shows more synergy variability compared to the healthy group and the alterations of muscle synergies can be described as a combination of reference synergies by four synergy coordination patterns. These findings deepen the understanding of the underlying neurophysiological mechanisms and possible motor control strategies adopted by the CNS in post-stroke patients.

## 1 Introduction

Recent studies in animals (Tresch et al., 1999; d’Avella et al., 2003a; Ting and Macpherson, 2005) and humans (Ivanenko et al., 2004; Weiss and Flanders, 2004; Torres-Oviedo and Ting, 2007a) reported that the central nervous system (CNS) coordinates a small set of motor modules, located in the motor cortex and spinal cord, commonly referred to as muscle synergies, to generate purposeful tasks. These modules are characterized as independent motor primitives with constant weight coefficients with respect to an ensemble of muscles (d’Avella et al., 2003b; Torres-Oviedo and Ting, 2007b; Bizzi et al., 2008). Coordinated recruitment and activation of these individual primitives by the cortical descending neural commands (i.e., temporal coefficients) in turn co-activates a set of synergies related to the movement. Muscle synergies largely simplify motor production (d’Avella et al., 2006; Bizzi and Cheung, 2013; Ting et al., 2015). However, cortical impairment disrupts descending neural commands from the brain (Cheung et al., 2009; Tropea et al., 2013), leading to modification of synergic patterns and abnormal recruitment of motor primitives and poor motor performances at the level of coordination across joints and range of motion (Cirstea et al., 2003; Beer et al., 2004).

Some studies have reported alterations of upper-limb muscle synergies in post-stroke patients in the number and structure compared to the control group. For example, Roh et al. reported shoulder-related changes of muscle synergies in mildly-to-severely impaired chronic stroke patients under upper-limb isometric force generation tasks (Roh et al., 2013; 2015), and the alteration was correlated with the impairment level and post-stroke duration (Cheung et al., 2012; Irastorza-Landa et al., 2021). Changes of muscle synergies were interpreted as the preservation, merging, or fractionation of synergies extracted from the control group (Clark et al., 2010; Cheung et al., 2012). High preservation of muscle synergies in chronic stroke patients without intact sensorimotor cortex was found, whereas patients with intact sensorimotor cortex showed poorer preservation and an increase in newly generated synergies (García-Cossio et al., 2014). Funato et al. (2022). verified the availability and relevance of muscle synergies in explaining physiological impairments by Fugl-Meyer (FM) Assessment Upper Extremity tasks. Further, our preliminary study (Zhao et al., 2022a) showed that post-stroke patients may adopt four synergy coordination patterns to control upper-limb tasks, including preservation, merging, sharing, and losing, in which sharing and losing were a refinement of the fractionation in Cheung’s work (Cheung et al., 2012). Specifically, sharing is a kind of special fractionation, which requires that all unaffected synergies are involved in explaining the affected synergies, and losing indicates that some unaffected synergies do not participate in the reconstruction of the affected synergies. Overall, these findings reported the relevance of lesion location, emerging descending pathways, and regulation in shaping muscle synergies (McMorland et al., 2015) and revealed possible modulation mechanisms adopted by the CNS to compensate for motor deficits after brain lesions.

Despite the promising results when using muscle synergies as physiological markers to assess motor function (Wang et al., 2020; Sheng et al., 2022), muscle synergies have not been fully investigated and widely used in clinical scenarios (Zhao et al., 2023), and there is room for more evidence as how to use synergies as biomarkers is still an open question, as a few evidence are available and not always in agreement. Besides some subjective factors, such as experimental protocols and synergy analysis methods (Steele et al., 2013; Banks et al., 2017; Zhao et al., 2022b; Zhao et al., 2022c), individual variability and subject- and task-specific muscle synergies make the outputs difficult to generalize and compare across studies (Zhao et al., 2021; Cartier et al., 2022). To promote the use of muscle synergy analysis in clinical assessment and personalized therapy and deepen the understanding of the pathological mechanisms in post-stroke patients, this study focused on the alterations of synergy structure in post-stroke patients by synergy reconstruction analysis and hypothesized that stroke patients adopted specific synergy coordination patterns for upper-limb motor control as described in previous work (Cheung et al., 2012) and coordination patterns were associated with the motor function.

To this end, a cohort of post-stroke patients with mild-to-moderate impairment and age-matched healthy subjects was recruited to perform two upper-limb functional tasks for muscle synergy analysis. A set of reference synergies (averaged across healthy subjects) and typical synergies (averaged across stroke subjects) were first extracted from healthy and stroke subjects, respectively. Muscle synergies extracted from stroke and healthy subjects and synergy coordination patterns were compared and analyzed at the group and individual levels. This study verified that, as previously hypothesized, four synergy coordination patterns (merging, sharing, losing, and preservation) can account for post-stroke patients’ synergies under the current experimental setups, and preservation was mainly represented in the mild impairment group and moderate impairment group showed more merging and sharing.

## 2 Materials and methods

### 2.1 Participants

Twenty post-stroke patients participated in the experiment but only thirteen individuals (age 40–72 years; 7 males) finished all tasks. Twenty neurologically intact age-matched healthy subjects (age 47–73 years; 11 males) were recruited as the control group in this study. The demographics of the participants was shown in Table 1. All participants were informed of the experimental procedure and provided written informed consent. The experiments were performed in accordance with the Declaration of Helsinki. The study was approved by the Ethics Committee of the Affiliated Jiangsu Shengze Hospital of Nanjing Medical University.

**TABLE 1:** Participant demographics of stroke and control groups.

	Mean	SD	Range
**Mild impairment group (P1-P7)**	—	—	—
Age (year)	60.7	9.9	40–68
Time after stroke onset (month)	5.7	4.6	0.5–13
Fugl-Meyer (/66)	58.7	3.3	54–63
Sex (M/F)	—	—	4/3
Side affected (L/R)	—	—	4/3
**Moderate impairment group (P8-P13)**	—	—	—
Age (year)	60.8	7.9	49–72
Time after stroke onset (month)	3.9	6.9	0.5–18
Fugl-Meyer (/66)	42.8	3.2	39–47
Sex (M/F)	—	—	3/3
Side affected (L/R)	—	—	3/3
**Healthy group**	—	—	—
Age (year)	57.5	7.8	47–73
Sex (M/F)	—	—	11/9

### 2.2 Experimental protocol and EMG recordings

Considering the importance of multi-joint tasks in daily activities, and requirements of variability and functionality of assessment tasks in muscle synergy analysis in the clinic, reaching movement (RM) and hand-to-mouth movement (HtMM) were evaluated in this study (Caimmi et al., 2015). These two tasks have been canonical paradigms (Levin et al., 2004) and have largely been used in clinical and laboratory research (Scano et al., 2018).

**FIGURE 1:**
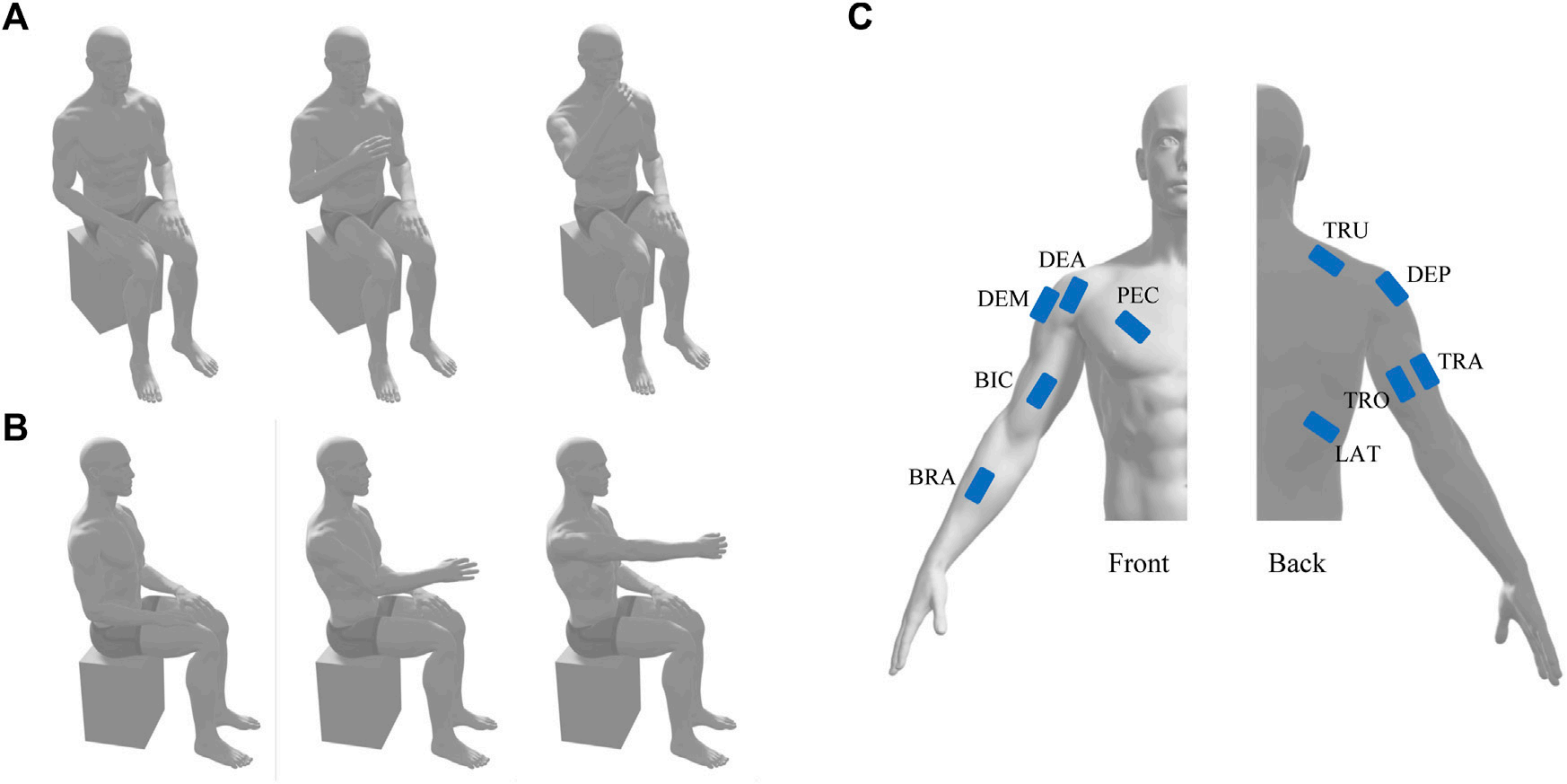
Schematic diagram of hand-to-mouth movement **(A)** and reaching movement **(B)** and the position of EMG electrodes **(C)**. TRO and TRA, triceps brachii long and lateral head; LAT, latissimus dorsi; PEC, pectoralis major; DEA, DEM, and DEP, deltoid anterior, medial, and posterior; TRU, trapezius upper; BIC, biceps brachii; BRA, brachioradialis.

Both tasks were performed in the sitting position (Figures 1A, B). Participants placed their hands on the thigh with upper-limb relaxation, then performed the movement in a self-selected rhythm. For the HtMM, the task consisted in reaching the mouth with the hand (or the possible closest position) and going back to the initial position. For the RM, the task was to reach 90° for shoulder flexion or reach the farthest position, and go back to the initial position. Compensatory movement of the torso was discouraged during movements. Given the strength and endurance of patients, each movement was repeated three times with a 2–3 s time interval. Three trials for each task were considered, as it was the minimum number that all patients could perform. A 30-s rest between tasks was followed to avoid muscle fatigue.

Even though motor deficits were also observed in the unaffected side of stroke patients (Selvarajan et al., 2019) and the existence of distributed mechanisms of synergic control in hand dominance (Madarshahian and Latash, 2022), the changes of muscle synergies are more evident in the affected side. Thus, this study recorded surface electromyography (EMG) signals from the dominant side of healthy subjects or the affected side of patients. Ten surface electrodes (Trigno Wireless EMG System, Delsys, United States) were placed on the muscle belly according to the Surface Electromyography Guidelines for the Non-invasive Assessment of Muscles, including a unilateral set up with the triceps brachii long and lateral head (TRO and TRA), latissimus dorsi (LAT), pectoralis major (PEC), deltoid anterior, medial, and posterior (DEA, DEM, and DEP), trapezius upper (TRU), biceps brachii (BIC), and brachioradialis (BRA) (Figure 1C). Another electrode that could simultaneously measure muscle activities and three-dimension acceleration was placed near the wrist for future data segmentation. EMG signal and acceleration signal was sampled at 1926 Hz and 148 Hz, respectively.

### 2.3 Signal preprocessing and synergy extraction

Acceleration data of the wrist were first smoothed for segmenting the trials. The onset and offset of each trial were defined as the points at which the net acceleration was below a threshold. EMG data were then extracted and preprocessed offline. Raw EMG signals were band-pass filtered at 20–450 Hz (fourth-order Butterworth filter), detrended, rectified, low-pass filtered at 5 Hz (fourth-order Butterworth filter), integrated over 20 ms, and resampled. To decrease the influence of amplitude differences and ensure that the extraction of muscle synergies was not biased against low-amplitude muscles, the envelope of each muscle and trial was normalized by unit variance (Roh et al., 2015).

Muscle synergies were extracted from a pooled EMG matrix ( $M\in R_{+}^{600\times 10}$ , 10 muscles, 2 tasks, 3 trials of each task, and 100 samples of each trial), including all trials and tasks of each individual. The non-negative matrix factorization (NMF) algorithm (Lee and Seung, 1999; Lee and Seung, 2001) was used to extract muscle synergies. An initial input indicating the number of synergies (one to ten in this study) is required to perform the algorithm. NMF factorizes the muscle activation matrix into a time-invariant weight matrix (muscle synergy) and a time-variant activation profile matrix. The extraction was iterated 50 times to avoid a local optimal solution.

The variance accounted for (VAF) (Roh et al., 2013; Zhao et al., 2019) was computed for each initial input to determine the optimal number of synergies required to reconstruct the variation of the original muscle activation. The optimal number of synergies was defined as the point at which the VAF value was above 95% (Tang et al., 2017; Pan et al., 2018).

### 2.4 Data analyses

#### 2.4.1 Synergy similarity

Synergy similarity was used to assess the similarity level among muscle synergies. For two muscle synergy matrices $W_{1}$ and $W_{2}$ (with *m* and *n* synergies respectively and m≤n ), synergy similarity was computed as follows (Tang et al., 2017):

$$Sim\left(W_{1},W_{2}\right)=\frac{1}{m}\sum_{i=1}^{m}\max\left[r\left(w_{1}^{i}∙w_{2}^{j}\right){|}_{j=1}^{n}\right]$$ (1)

where $w_{1}^{i}$ and $w_{2}^{j}$ are the *i*th and *j*th synergy vector of $W_{1}$ and $W_{2}$ , respectively. $r\left(w_{1}^{i}∙w_{2}^{j}\right)$ indicates the normalized scalar product of $w_{1}^{i}$ and $w_{2}^{j}$ .

#### 2.4.2 Reference and typical synergies

To avoid the effect of module dimensionality in building reference synergies, the same number of synergies was extracted from healthy subjects (Roh et al., 2013; Tropea et al., 2013; Pan et al., 2018; Irastorza-Landa et al., 2021). Synergies were matched by similarity (normalized scalar product) across subjects and then averaged, to achieve reference synergies. We further performed the aforementioned procedure in the stroke groups (mild impairment group and moderate impairment group), and obtained a set of typical synergies which indicated the synergy characteristics of each group. Synergy similarity between groups was computed to evaluate inter-group similarity.

#### 2.4.3 Synergy reconstruction analysis

**FIGURE 2:**
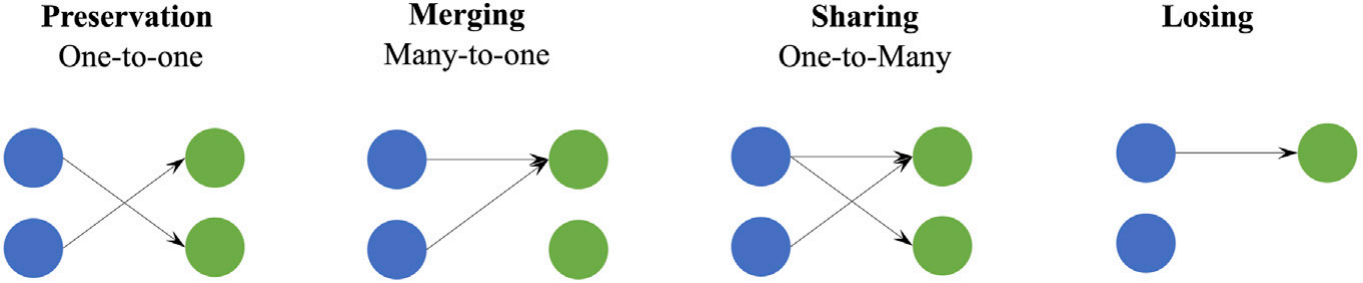
Schematic of four synergy coordination patterns, preservation, merging, sharing, and losing. Blue circles represent the reference synergies and green circles are the affected synergies. For preservation, merging, and sharing, all reference synergies are involved in the generation of affected synergies, and they are summarized as one-to-one, many-to-one, and one-to-many, respectively.

Reference synergies were regarded as a set of basic synergy primitives, which characterized normal coordination patterns of the CNS in controlling HtMM and RM. We assumed that CNS lesions could affect the recruitment of this set of modules in number and/or composition and cause abnormal synergy coordination patterns that can be explained by preservation, losing, merging, and sharing as in previous works (Cheung et al., 2012; Zhao et al., 2022a) (Figure 2). The preservation takes place when all reference synergies participate in the reconstruction of affected synergies, and each reference synergy corresponds to one affected synergy. The losing indicates that some reference synergies do not participate in the reconstruction of affected synergies, i.e., the reconstruction coefficient is below a threshold. In terms of the merging, all reference synergies are involved in the reconstruction of the affected synergies, and at least one affected synergy is reconstructed by multiple reference synergies. Finally, the sharing means that all reference synergies participate in the reconstruction of affected synergies, and at least one reference synergy simultaneously contributes to multiple affected synergies. Our aim is to determine which of these patterns are used by patients and if they relate to their clinical conditions.

To evaluate synergy coordination patterns adopted in stroke patients, we reconstructed affected or typical synergies by synergy merging and fractionation analyses (Clark et al., 2010; Cheung et al., 2012; Pan et al., 2018). Synergy merging hypothesizes that affected synergies could be explained as a linear combination of reference synergies. Synergy fractionation assumes that affected synergies are from the fractionation of one reference synergy.

$$w_{A}\approx W_{R}*c_{M}$$ (2)

$$w_{R}\approx W_{A}*c_{F}$$ (3)

where $W_{R}$ and $w_{R}$ denote reference synergy matrix and each synergy component, respectively. $W_{A}$ and $w_{A}$ are affected/typical synergy matrix and each synergy component, respectively. $c_{M}$ and $c_{F}$ indicates reconstruction coefficients (merging and fractionation coefficients), which were computed by a non-negative least-squares algorithm. The reconstruction procedure was regarded as valid if the reconstruction coefficient was above 0.3 as previously proposed (Barroso et al., 2014; Pan et al., 2018). The similarity between the reconstructed synergy and those extracted from patients was computed using the normalized scalar product to evaluate the reconstruction quality. If reconstruction similarity was above 0.7 (Cheung et al., 2012; Barroso et al., 2014), the reconstructed synergy could represent the original one. Considering that one affected synergy could not be the fractionation of multiple reference synergies, the fractionation with a maximum reconstruction coefficient was effective when several reference synergies contributed to one affected synergy, that is if there are multiple reconstruction coefficients with a value above 0.3, the largest one is chosen.

#### 2.4.4 Statistical analyses

The ANOVA was used to evaluate statistical differences in the number of synergies and synergy similarity among groups. All procedure was performed in Matlab R2020b. The significance level was set at 0.05.

## 3 Results

### 3.1 Number of muscle synergies

**FIGURE 3:**
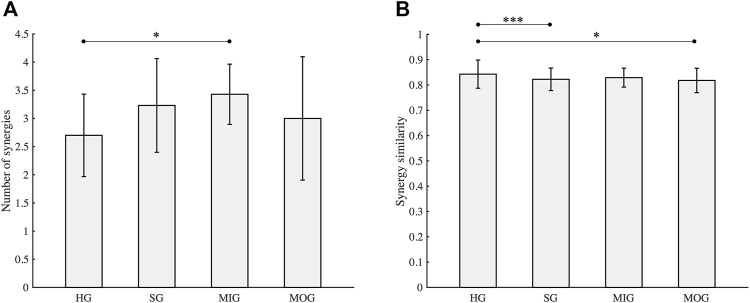
Number of synergies **(A)** and synergy similarity **(B)**. HG, healthy group; SG, stroke group; MIG, mild impairment group (FM ≥ 50); MOG, moderate impairment group (FM < 50). Asterisks (*, ***) indicate significance level (0.05, 0.001).

Thirteen mild-moderate post-stroke patients completed both tasks. According to the criteria used to identify the number of synergies, 2.7 ± 0.73 and 3.2 ± 0.83 (mean ± SD) synergies were extracted from the healthy group (HG) and stroke group (SG), respectively. Although the mean number of synergies extracted from the stroke group was slightly larger than that of the healthy group (Figure 3A), no statistical difference was found [F(1, 30) = 3.72, *p* = 0.06]. In addition, when the same number of muscle synergies was extracted from both groups, the VAF of the healthy group was above that of the stroke group. The stroke group was further divided into mild impairment group (MIG, FM ≥ 50) and moderate impairment group (MOG, FM < 50). The number of synergies of the MIG and MOG was compared with the HG, respectively. The results showed that the number of synergies of HG and MOG had no statistical difference (2.7 ± 0.73 vs. 3.0 ± 1.10, F(1, 23) = 0.62, *p* = 0.44), while a significantly higher number of synergies was found in MIG (3.4 ± 0.53 vs. 2.7 ± 0.73, F(1, 24) = 5.78, *p* = 0.02). Statistical difference in the number of synergies was not observed between MIG and MOG (F(1, 10) = 0.85, *p* = 0.38).

### 3.2 Synergy similarity analysis

Similarity analysis (Figure 3B) showed that the mean similarity of the healthy group was the highest (0.84 ± 0.06), followed by the mild impairment group (0.83 ± 0.04). The similarity of the stroke group (0.82 ± 0.04) which includes all stroke subjects was comparable with the moderate impairment group (0.82 ± 0.05). Statistical analysis showed that the similarity of the healthy group was significantly higher than the stroke group and the moderate impairment group (*p* < 0.05), while a significant difference was not observed between the healthy group and the mild impairment group (*p* = 0.12).

### 3.3 Reference and typical synergies

**FIGURE 4:**
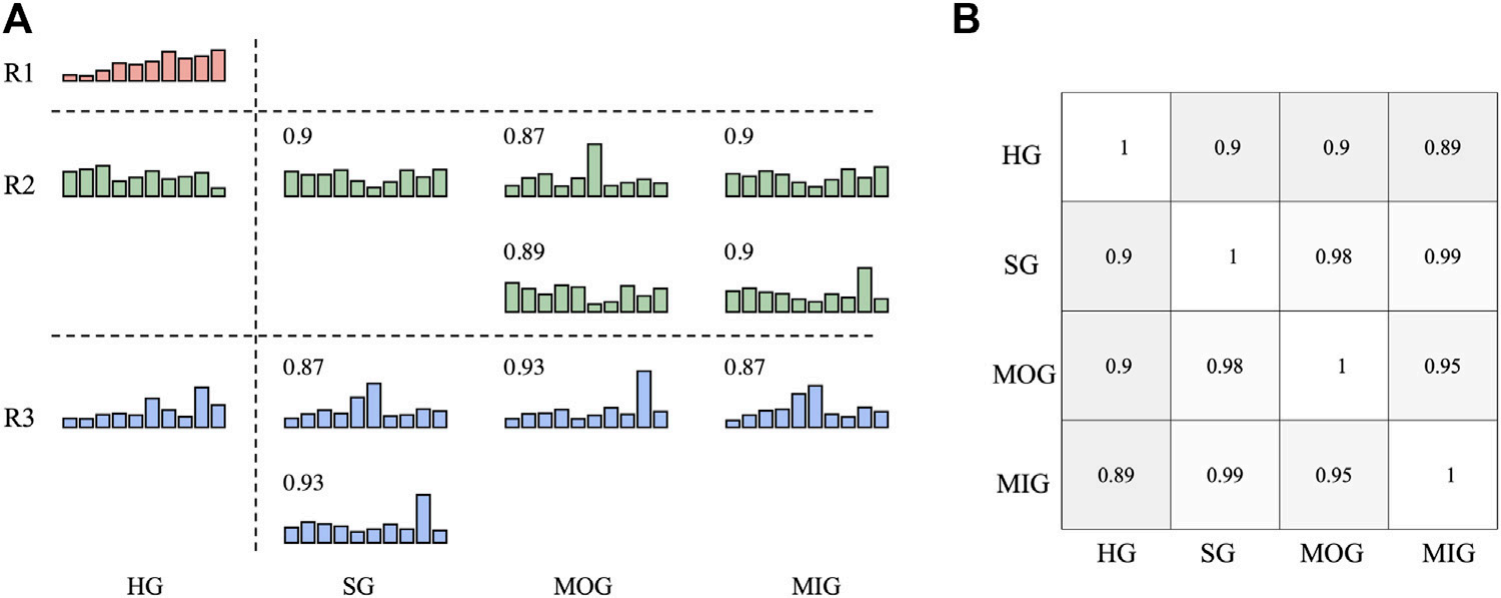
Reference synergies and typical synergies **(A)**. The number is the synergy vector similarity between reference synergies and typical synergies of each group is shown. **(B)** Shows the inter-group synergy similarity.

The reference synergies and typical synergies of each group are shown in Figure 4. We noticed that SG, MOG, and MIG “lost” the reference synergy R1, and there were two typical synergies “sharing” one reference synergy in each group (Figure 4A). A high inter-group synergy similarity was observed (Figure 4B).

### 3.4 Synergy reconstruction analysis at the group level

**FIGURE 5:**
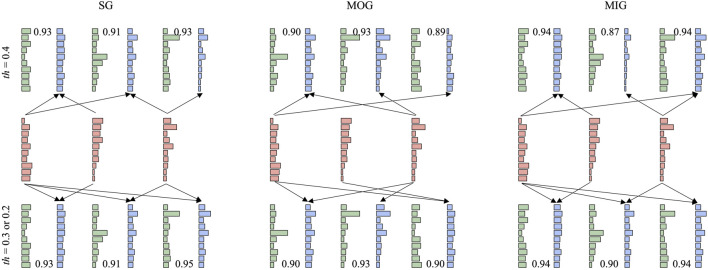
Synergy reconstruction at the group level. Three thresholds (0.2, 0.3, and 0.4) were set for synergy reconstruction analysis. The first row shows the results with a threshold of 0.4. The second row shows reference synergies. When the threshold is 0.2 or 0.3, the reconstruction results are very similar (the third row). Red, green, and blue bars represent reference synergies, typical synergies, and reconstructed synergies, respectively. We showed the procedure (arrow) with a reconstruction coefficient above the threshold and reconstruction similarity.

**FIGURE 6:**
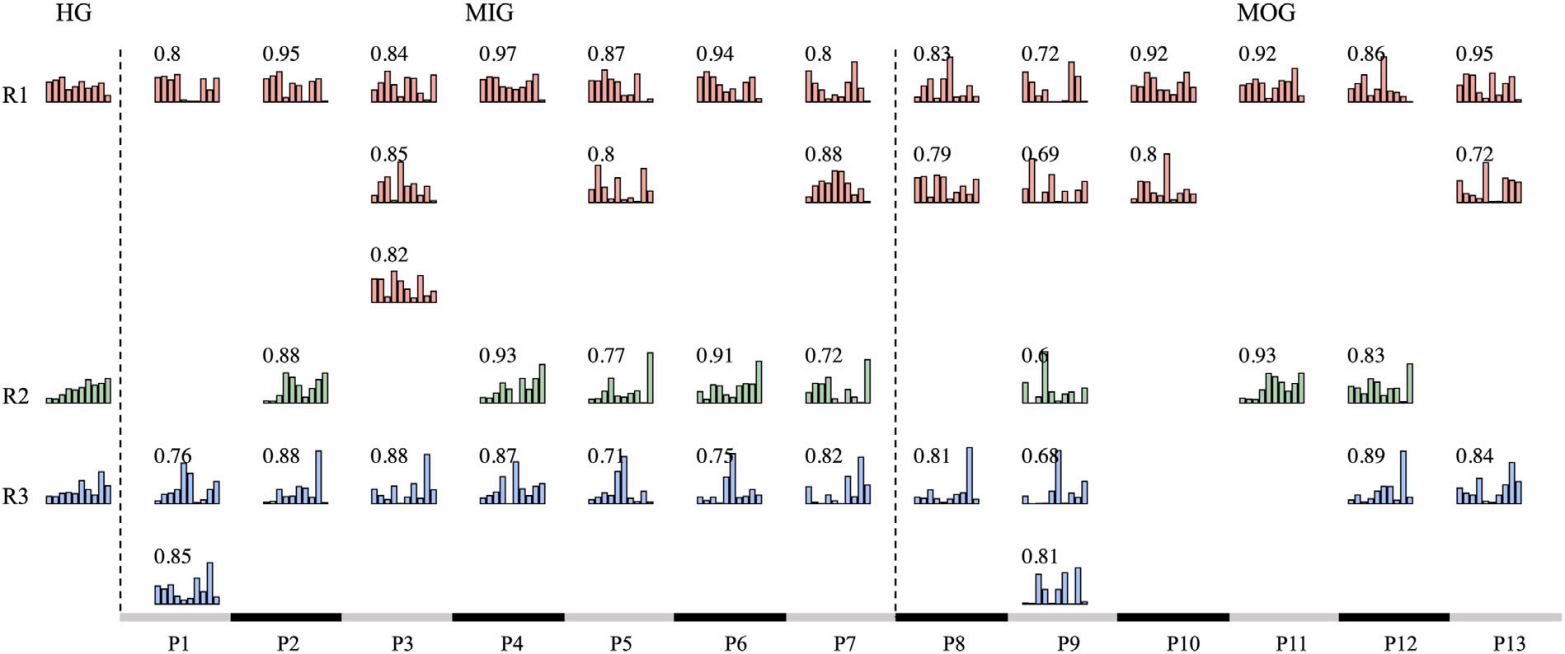
Synergy matching between synergies of each patient and reference synergies. The HG column represents reference synergies. MIG and MOG columns are the synergies of each patient. We paired the reference synergies and patients’ synergies by scalar product. Similarity values are shown in each subplot.

Synergy reconstruction analysis was first performed at the group level (Figure 5), i.e., typical synergies were reconstructed by reference synergies. All three reference synergies were involved in the reconstruction, and reconstruction similarities were all above 0.9 when the reconstruction coefficient threshold was 0.2 or 0.3. Then, we paired the synergies of each patient and reference synergies (Figure 6) and found that several reference synergies were “lost” in patients (e.g., P11), or one reference synergy was “shared” by multiple affected synergies (e.g., P3, P5, P8), or three reference synergies corresponded to the affected synergies one-by-one, that is, synergies were “preserved” (e.g., P2, P4, P6).

### 3.5 Synergy reconstruction analysis at the individual level

**FIGURE 7:**
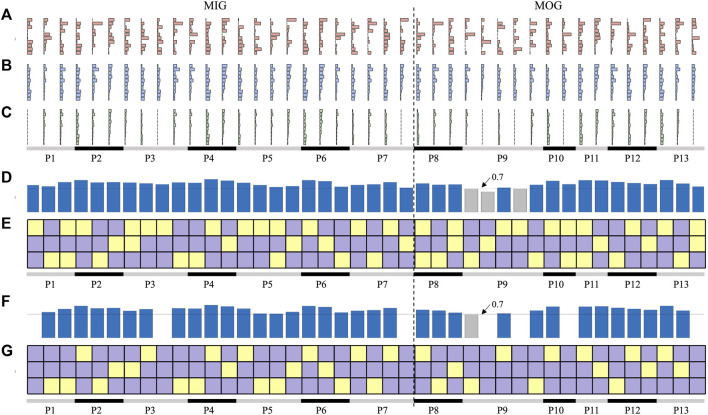
Synergy reconstruction analysis at individual level. **(A)** Synergies of patients (affected synergies). **(B, C)** show the reconstructed synergies by merging and fractionation analyses. **(D, E)** is the reconstruction similarity and coefficients. In **(E, G)**, three squares in each column indicate the reconstruction coefficient when three reference synergies were used to reconstruct each affected synergy. Yellow squares represent the reconstruction coefficient above 0.3, and purple squares are below 0.3. The bars in **(D, F)** indicate the reconstructed similarity corresponding to each affected synergy. Blue bars represent the reconstruction similarity above 0.7 and gray bars below 0.7.

We further performed synergy reconstruction analysis at the individual level (Figure 7). For each individual, each affected synergy can be reconstructed by a linear combination of one to two reference synergies (Figures 7B, E). Some patients (P2, P4, P6, and P12) had healthy-like synergies in number and structure, i.e., three synergies were identified and can be respectively reconstructed by three reference synergies with a reconstruction similarity above 0.7 (Figure 7D). Except for P7 who extracted five synergies, in which three reconstruction similarity was below 0.7, all patients had reconstructed similarity above 0.7.

Considering that not all reconstructed synergies had a reconstruction similarity above 0.7, we performed reconstruction analyses by synergy fractionation (Figure 7C). Even though most of the affected synergies can be described as the fractionation of one reference synergy, there were several affected synergies that cannot be reconstructed (Figures 7F, G) when the reconstruction coefficient was set 0.3. In addition, there was an overlap between synergy merging and fractionation processes, that is, one affected synergy could be reconstructed by synergy merging and fractionation of the same reference synergy. For example, three affected synergies of P2 were regarded as the linear combination of three reference synergies (Figure 7E), and these synergies can also be reconstructed by factorizing three reference synergies respectively (Figure 7G), and reconstruction similarity under these two conditions was all above 0.7. This often appeared in patients who had healthy-like synergies.

### 3.6 Synergy coordination patterns in post-stroke patients

**FIGURE 8:**
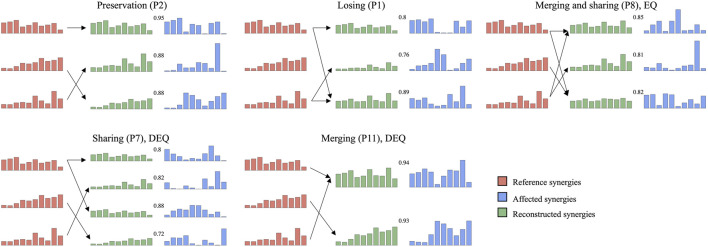
Representative subjects of each muscle synergy coordination pattern.

In summary, affected synergies could often be explained as a linear combination of reference synergies, and four synergy coordination patterns in post-stroke patients were shown: preservation, losing, merging, and sharing. According to whether the number of synergies of patients was equal to the number of reference synergies, merging and sharing patterns were further divided into equilibrium (EQ) and disequilibrium (DEQ) modes. The EQ means that the number of synergies is equal to three, instead, the DEQ has more or fewer synergies (Table 2; Figure 8).

**TABLE 2:** Synergy coordination patterns. NoS, number of synergies. EQ, equilibrium. DEQ, disequilibrium.

		P1	P2	P3	P4	P5	P6	P7	P8	P9	P10	P11	P12	P13
NoS		3	3	4	3	4	3	4	3	5	2	2	3	3
Preservation			✓		✓		✓						✓	
Losing		✓									✓			
Merging	EQ	✓							✓					✓
	DEQ			✓		✓					✓	✓		
Sharing	EQ	✓							✓					✓
	DEQ			✓		✓		✓		✓	✓			

The results showed that the preservation was mainly in the mild impairment group (3/4), and only one moderately impaired patient showed preservation. The moderate impairment group showed more merging and sharing. Meanwhile, a small group of individuals (P1, P8, and P13) showed the equilibrium mode of merging and sharing. More than half of the individuals represented disequilibrium mode in merging and sharing groups. One individual showed losing in both mild and moderate impairment groups.

**FIGURE 9:**
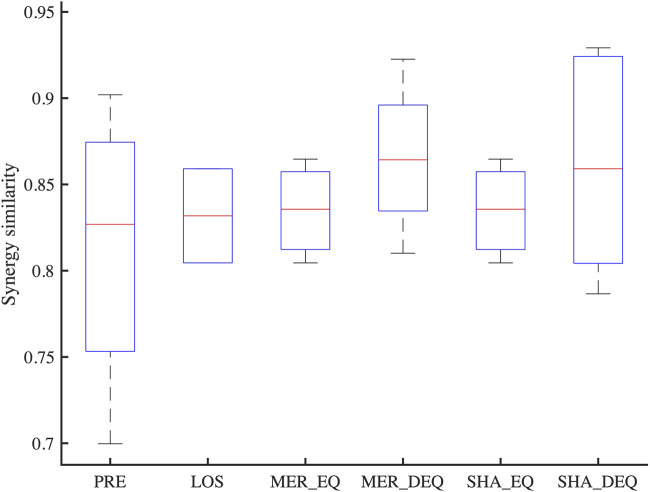
The similarity between affected synergies of each synergy coordination pattern and reference synergies. PRE, Preservation; LOS, Losing; MER_EQ, Equilibrium mode of merging; MER_DEQ, Disequilibrium mode of merging; SHA_EQ, Equilibrium mode of sharing; SHA_EQ, Disequilibrium mode of sharing.

Synergy similarities between affected synergies of each synergy coordination pattern and reference synergies are shown in Figure 9. Statistical differences were not observed across patterns [F(3, 16) = 0.52, *p* = 0.68]. The post-hoc test also did not show a significant difference between patterns (*p* > 0.05).

## 4 Discussion

This study analyzed muscle synergies of two representative groups of mild and moderate post-stroke patients in two upper-limb functional tasks. Based on the reference synergies extracted from healthy subjects, this study confirmed four muscle synergy coordination patterns adopted by the CNS in stroke patients to adapt and compensate for abnormal motor performances.

Previous evidence showed a decrease in the dimensionality and complexity of muscle synergies after stroke (Cheung et al., 2012; García-Cossio et al., 2014; Tang et al., 2017). However, in this study we selected two assessment tasks (RM and HtMM) accessible for subjects after stroke and, on average, three synergies were extracted from both groups. Patients with fewer synergies (2/13) were all from the moderately impaired group, while occasionally more synergies emerged in the mild impairment group (4/13). The results indicated that moderate impairment patients might use fewer synergies to control RM and HtMM. This finding is consistent with the fact that fewer synergies may underlie poor control in independent functionality tasks. However, we have to note that this study evaluated two upper-limb multiple-joint functionality tasks, that explore functional movements toward a target (RM) and toward the body (HtMM) but limit motor variability with respect to previous works (Roh et al., 2013). Thus, a relatively low number of synergies was identified. A similar number of synergies were also reported under various upper-limb tasks (Roh et al., 2013; Tropea et al., 2013), indicating that extracted synergies sufficiently captured variations in muscle activation resulting from maladaptive and compensatory mechanisms of the CNS in motor control (Scano et al., 2017).

Stroke can cause alternations of muscle synergies in number, structure, or recruitment patterns (Pierella et al., 2020; Irastorza-Landa et al., 2021; Park et al., 2021), and the changes in synergy structure can be described by merging and fractionation of the synergies extracted from the control group (Cheung et al., 2012). Moreover, the alternation of muscle synergies in post-stroke patients served for motor deficits with a compensatory strategy of the CNS (Gizzi et al., 2011; Scano et al., 2017), which is intentional and spontaneous rather than random. Thus, this study reasonably assumes that before the event, the muscle synergies of patients were similar to the control group and used control synergies as a reference, and verified that synergy similarity is lower in stroke groups. In detail, our analyses verified that the highest synergy similarity was observed in the healthy group and that the moderate impairment group had a lower similarity compared with other groups. Inter-group similarity over the individual level also showed a high similarity value. The robustness of the synergy structure indicated that the CNS flexibly recruited a set of muscle synergies located in the spinal cord or/and brainstem to drive movements, even though stroke patients had abnormal performance (Cheung et al., 2009).

Although some studies have reported that disease or neurorehabilitation training can induce the generation of novel synergies or changes in synergy weights (Coscia et al., 2019), there are still no consistent conclusions on how muscle synergies change in post-stroke patients. By synergy reconstruction analysis, this study confirmed that muscle synergies of patients mainly presented a linear combination of reference synergies. Finally, four muscle synergy coordination patterns in mild-moderate stroke patients were shown based on models introduced in previous works (Cheung et al., 2012), in which three synergy coordination patterns, preservation, merging, and fractionation, were proposed to quantify alterations of muscle synergies. Preservation was found mainly in the mild impairment group, and the moderate impairment group showed more merging and sharing. Actually, stroke interferes with the pathway from the brain to the spine, causing poor motor performance, which can be explained by reduced or abnormal independent motor modules by muscle synergy analysis (Bizzi et al., 2008). Thus, this work confirmed previous findings that showed that synergy patterns might be related to the severity of motor impairment. Although there were no evident results in terms of the patterns within and between groups, the current four synergy coordination patterns, merging (equilibrium and disequilibrium), sharing (equilibrium and disequilibrium), losing, and preservation, extended previous findings, which deepens our knowledge in understanding the neurophysiological mechanisms and possible control strategies adopted by the CNS in stroke patients. Besides, the patients with equilibrium mode are more focused on the early stage of stroke (1–3.5 months), while a wide range of onset time from 0.3 to 18 months was reported in the disequilibrium group in this study. We conjecture that equilibrium and disequilibrium modes might reflect the transition of disease among stages, which can be novel insight to assess the status of the patients. However, due to the limitation of the number of participants, this study does not conclude significant results about the physiological mechanism of equilibrium and disequilibrium synergy patterns. More patients are needed for further study.

We have to note that the current results were based on the reconstruction coefficient at 0.3 as shown in previous works (Cheung et al., 2012; Barroso et al., 2014; Pan et al., 2018). This study showed that the selection of the reconstruction coefficient affected the results of synergy reconstruction analysis (Figure 5). When a larger threshold was selected, few affected synergies can be reconstructed by reference synergies. On the other hand, when the threshold was small, the affected synergies might be regarded as the combination of all reference synergies, which results in large redundancy and makes the results difficult to explain. Whatever, four synergy coordination patterns can always be found. In summary, losing might appear more if the threshold is higher, instead, a lower threshold might cause more merging and sharing. Even though the results are limited to the current experiment setups, we still conclude some promising and meaningful results.

This study has some limitations. First, only thirteen mildly to moderately impaired patients finished the tasks. Thus, current results lack a description of severely impaired patients, which might have different synergy patterns. Moreover, this study only assessed two upper-limb functionality gestures, which reflect limited variability of motor space, causing fewer muscle synergies compared to previous studies. In addition, four synergic patterns were found, while some patients simultaneously exhibited merging and sharing patterns. It is still an open question if these patterns underlie synergy coordination patterns in post-stroke patients and how these results would be affected by a higher movement variability and more samples. Due to the limitations of the number and diversity of the recruited patients and experimental protocol (tasks and constraints) and methods adopted to extract and analyze muscle synergies, the current study only proposes a possible hypothesis that is usable in clinical scenarios, and further investigations are required in future work.

## 5 Conclusion

This study concluded that muscle synergies extracted from post-stroke patients can be described as a linear combination of a set of reference synergies. Four synergic patterns adopted by the CNS in a cohort of stroke patients with mild-to-moderate impairment were described: preservation, losing, merging, and sharing, and synergic patterns were associated with the motion function. The results would contribute to the application of muscle synergies in patient-specific rehabilitation assessment.

## Data Availability

The raw data supporting the conclusion of this article will be made available by the authors, without undue reservation.
